# Gaze-dependent spatial updating of tactile targets in a localization task

**DOI:** 10.3389/fpsyg.2014.00066

**Published:** 2014-02-10

**Authors:** Stefanie Mueller, Katja Fiehler

**Affiliations:** Department of Psychology, Justus-Liebig University GiessenGiessen, Germany

**Keywords:** movement, reference frames, spatial localization, spatial updating, tactile, visual

## Abstract

There is concurrent evidence that visual reach targets are represented with respect to gaze. For tactile reach targets, we previously showed that an effector movement leads to a shift from a gaze-independent to a gaze-dependent reference frame. Here we aimed to unravel the influence of effector movement (gaze shift) on the reference frame of tactile stimuli using a spatial localization task (yes/no paradigm). We assessed how gaze direction (fixation *left/right*) alters the perceived spatial location (point of subjective equality) of sequentially presented tactile standard and visual comparison stimuli while effector movement (gaze *fixed/shifted*) and stimulus order (*vis-tac/tac-vis*) were varied. In the fixed-gaze condition, subjects maintained gaze at the fixation site throughout the trial. In the shifted-gaze condition, they foveated the first stimulus, then made a saccade toward the fixation site where they held gaze while the second stimulus appeared. Only when an effector movement occurred *after* the encoding of the tactile stimulus (shifted-gaze, *tac-vis*), gaze s*imilarly* influenced the perceived location of the tactile and the visual stimulus. In contrast, when gaze was fixed or a gaze shift occurred *before* encoding of the tactile stimulus, gaze *differentially* affected the perceived spatial relation of the tactile and the visual stimulus suggesting gaze-dependent coding of only one of the two stimuli. Consistent with previous findings this implies that visual stimuli vary with gaze irrespective of whether gaze is fixed or shifted. However, a gaze-dependent representation of tactile stimuli seems to critically depend on an effector movement (gaze shift) after tactile encoding triggering spatial updating of tactile targets in a gaze-dependent reference frame. Together with our recent findings on tactile reaching, the present results imply similar underlying reference frames for tactile spatial perception and action.

## Introduction

Object locations in our environment can be derived through various sensory channels which end in sensory-specific spatial maps. One inherent complexity arises when spatial information represented in different coordinate systems needs to be compared for future action. For example, directing the hand toward a glowing object in the dark requires the spatial comparison of the locations of the effector (the hand) derived through proprioception and the object derived through vision. So far, it is still an open question which reference frames are used to localize and compare spatial information of different sensory modalities.

Previous studies suggest the use of a gaze-dependent reference frame when people are asked to localize a visual target in space. Bock (1986) first found that participants overestimate the remembered location of peripherally viewed visual targets; an effect called the retinal magnification effect (RME). They point to far to the right if gaze is directed left of the target and vice versa. Similar gaze-dependent errors were later reported when participants initially foveate the target and then shift gaze to an eccentric location after the target was extinguished (Henriques et al., 1998). It has been argued that the mental representation of the (remembered) visual target had been updated, or remapped, into the visual periphery due to the gaze shift and that this remapped target representation is used to plan the movement resulting in the RME. Gaze-dependent spatial updating of visual targets has consistently been observed for goal-directed reaching (e.g., Medendorp and Crawford, 2002; Beurze et al., 2006; Schütz et al., 2013) and grasping (Selen and Medendorp, 2011) as well as spatial localization tasks where participants judged the position of an eccentric (remembered or present) visual target with respect to a proprioceptive (Fiehler et al., 2010), visual (Eggert et al., 2001) or auditory (Lewald and Ehrenstein, 1996, 1998) comparison stimulus. This suggests similar spatial coding mechanisms for action and perception.

An influence of gaze on spatial localization has also been found for somatosensory targets; however, the findings are less consistent compared to visual targets. Behavioral studies demonstrated gaze-dependent reach (Pouget et al., 2002b; Blangero et al., 2005; Jones and Henriques, 2010; Reuschel et al., 2012) and localization errors (Harrar and Harris, 2009, 2010; Fiehler et al., 2010; Pritchett et al., 2012) for proprioceptive and tactile targets as obtained in experiments with visual targets. This may imply similar spatial coding mechanisms for visual and somatosensory target modalities. However, a neuroimaging study which examined the reference frames for visual and proprioceptive reach targets argued for a flexible use of gaze-centered and body-centered coordinate systems depending on the sensory target modality (Bernier and Grafton, 2010). More specifically, the authors suggest a dominant use of a gaze-dependent reference frame for visual targets and a gaze-independent, body-centered reference frame for proprioceptive targets.

In a recent study we showed that an effector movement probably accounts for the incongruent results reported in previous research on goal-directed reaching to proprioceptive targets (Mueller and Fiehler, 2013). We investigated whether reach errors varied as a function of gaze relative to target depending on the presence or absence of an effector movement before the reach. An effector movement could be either a gaze shift after target presentation and before reaching or an active movement of the target (non-reaching) arm to the target location. The movement conditions were compared with a stationary condition where gaze was fixed throughout the trial and the target arm remained at the target location. We observed gaze-dependent reach errors only in conditions where an effector movement was introduced before the reach. Thus, an effector movement (of the eyes or the target limb) seems to trigger a switch from a gaze-independent to a primarily gaze-dependent representation of somatosensory reach targets. We obtained this result for tactile and proprioceptive-tactile targets.

However, in our previous study (Mueller and Fiehler, 2013), the introduced effector movement of the target limb might have interfered with the reaching hand in contrast to the condition without effector movement. Dessing et al. (2012) recently suggested that the consistently observed gaze-dependent reach errors to visual targets originate (at least in part) from hand-related biases due to a misestimation of the proprioceptive feedback from the hand instead of a misestimation of the remembered target location (e.g., Henriques et al., 1998; Blohm and Crawford, 2007; Khan et al., 2007). Assuming that gaze-dependent reach errors are primarily caused by a mislocalization of the reaching hand, the question arises whether the influence of the effector movement on spatial coding, as observed in our previous study (Mueller and Fiehler, 2013), is merely due to interference of the introduced effector movement (eyes or arm) with the reaching hand. The goal of the present study was to test the influence of effector movement on gaze-dependent spatial coding of tactile targets in a perceptual localization task, thus eliminating the impact of reach-related localization errors of the hand. By applying a cross-modal approach, we were able to directly contrast the reference frames of tactile and visual stimuli while varying the presence or absence of an intervening effector movement.

We conducted a psychophysical spatial localization task (yes/no paradigm) where the remembered location of a tactile standard stimulus had to be judged relative to the location of a remembered visual comparison stimulus. By exploiting the profound evidence on gaze-dependent coding and updating of visual stimuli obtained in localization tasks (Lewald and Ehrenstein, 1996, 1998; Eggert et al., 2001; Fiehler et al., 2010), we aimed to unravel the underlying reference frames used to encode tactile location. Gaze was varied relative to a tactile standard (fixation: left or right) and held eccentric during the response. We further included two gaze conditions which differed by whether a gaze shift was introduced *between* the presentation of the tactile standard and the visual comparison (*shifted-gaze*) or gaze maintained fixed at an eccentric location *from the beginning* of the trial (*fixed-gaze*). The two gaze conditions were combined with two possible stimulus orders where the visual comparison was presented before the tactile standard (*vis-tac*) or vice versa (*tac-vis*).

The points of subjective equality (PSEs) were assessed as an indicator for the perceived spatial relation of the tactile and the visual stimulus. In particular, we examined how the PSEs varied as a function of gaze direction, thus allowing conclusions about the underlying gaze-dependent reference frame of the tactile stimulus. We would expect PSEs to vary similarly with fixation if both the tactile and the visual stimulus were represented in a gaze-dependent reference frame. In contrast, PSEs should vary differentially with respect to gaze if only the visual but not the tactile stimulus is coded in gaze-dependent coordinates.

**Figure 1:**
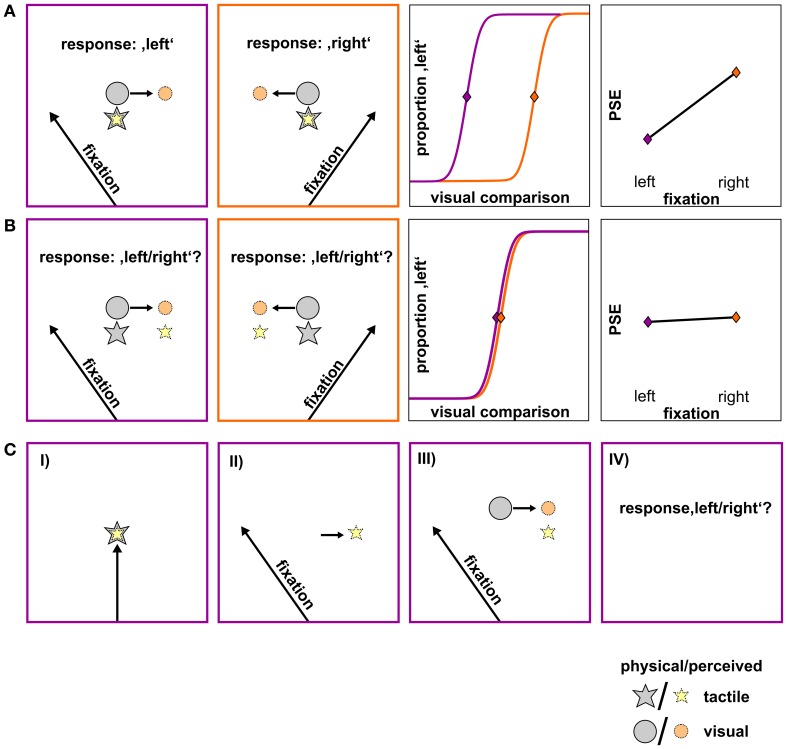
**Hypotheses on the influence of gaze direction on the spatial perception of the tactile and the visual stimulus, shown for a schematic standard-comparison combination. (A)** Differential influence of gaze. Fixation to the left or to the right of the stimuli causes the visual stimulus to be misestimated opposite to gaze direction (orange circle) while the tactile stimulus remains unaffected by gaze direction (yellow star). Consequently, the same spatial relation between the physical tactile (gray star) and the physical visual stimulus (gray circle) results in a perceived spatial relation (colored star/circle) that varies as a function of fixation (1st and 2nd panel). For a specific standard location to be perceived as aligned to a visual comparison, the visual comparison has to be presented more leftwards when the fixation is left and more rightwards when the fixation is right. This is reflected in a shift of psychometric functions and thus on PSEs depending on fixation (3rd and 4th panel; fixation left: lilac, fixation right: orange). **(B)** Similar influence of gaze. A gaze shift after the presentation of the tactile stimulus (not shown) causes its remembered location (yellow star) to shift by a similar gaze-dependent bias as the visual stimulus (orange circle) and thus, preserving their spatial relation (1st and 2nd panel). This is reflected in similar psychometric functions and according PSEs for left- and rightward gaze shifts (3rd and 4th panel; fixation left: lilac, fixation right: orange). **(C)** Trial sequence of the critical shifted-gaze, tac-vis condition where we expect a similar influence of gaze. (I) Presentation of the foveated tactile stimulus; (II) gaze shift to a peripheral fixation; gaze-dependent spatial update of the tactile stimulus; (III) presentation of the visual comparison; gaze-dependent spatial update of the visual stimulus; (IV) relation between the two stimuli is preserved and thus, the response does not change with fixation.

Figure 1 depicts the respective result patterns for the two potential outcomes. The first row (A) shows a *differential influence of gaze* direction on the visual and tactile stimulus. Here, we assume that the location of the (physical) visual stimulus (gray circle) is perceptually displaced opposite to gaze direction (orange circle) while the location of the (physical) tactile stimulus (gray star) remains unaffected by gaze direction (yellow star). Consequently, the (physical) visual comparison has to be presented farther left to be perceived as aligned with the tactile stimulus (=PSE) if the subject fixates to the left [row (A), 1st panel]. Conversely, if the subject fixates to the right, the perceived visual comparison is misestimated to the left and thus, the (physical) visual comparison has to be presented farther right to be perceived as aligned with the tactile stimulus [row (A), 2nd panel]. This finally leads to a divergence of PSEs as a function of fixation [row (A), 3rd and 4th panel]. The second row (B) depicts a *similar influence of gaze* on the tactile and the visual stimulus. Here, we assume that directing gaze to the left or right leads to a misestimation opposite to gaze for both the visual (orange circle) and the tactile (yellow star) stimulus [row (B), 1st and 2nd panel]. Thereby, the spatial relation between the two stimuli (which is reflected in the PSE) is preserved and should result in similar PSEs irrespective of fixation [row (B), 3rd and 4th panel].

Based on our previous findings (Mueller and Fiehler, 2013), we hypothesize that an effector movement (i.e., a gaze shift) which is executed after the presentation of the tactile standard leads to gaze-dependent spatial updating of the remembered tactile target. Note that this case depends on both gaze condition (shifted-gaze) *and* stimulus order (*tac-vis*). When the location of both the tactile standard and the visual comparison is updated with respect to gaze (orange circle), the spatial relation between the two stimulus modalities should be preserved resulting in similar PSEs (Figure 1B). In contrast, if no intervening gaze shift is present after the presentation of the tactile standard (i.e., in the conditions fixed-gaze, *vis-tac/tac-vis* and shifted-gaze, *vis-tac*) we hypothesize the tactile stimulus to be represented in a gaze-independent reference frame. Consequently, gaze direction should only affect the visual but not the tactile stimulus and thereby result in different PSEs varying as a function of fixation (Figure 1A).

## Methods

### Participants

Fifteen healthy participants took part in the experiment. After data cleaning (see section Data Analysis) the number of participants was reduced to 10 (males/females: 6/4, age range: 21–28 years, mean ± *SD*: 24 ± 2.4 years). All participants had normal or corrected to normal vision, were right handed, and provided written informed consent according to the local ethics committee. Course credits were received for participation.

### General experimental setup

**Figure 2:**
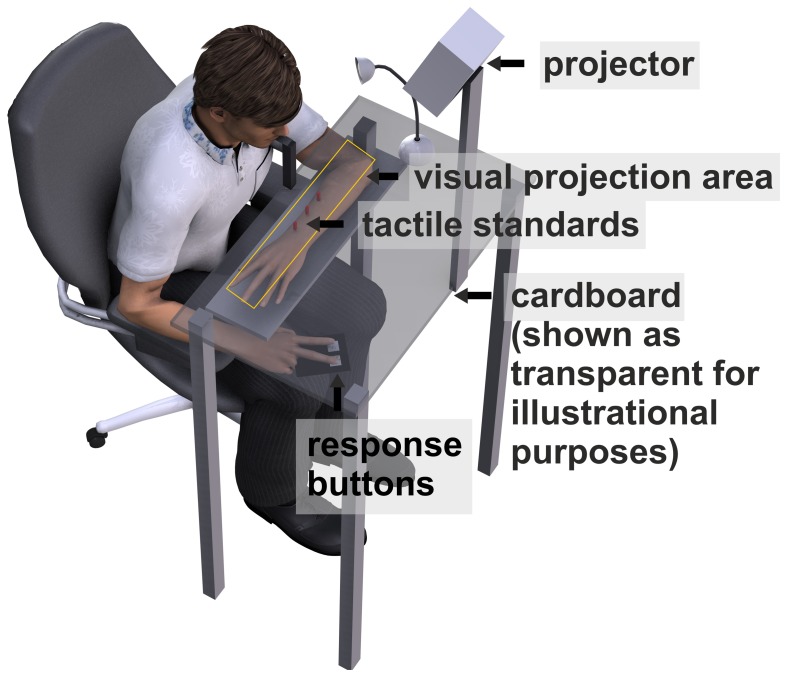
**Schematic of the apparatus**. Tactile standard stimuli were applied on the dorsal surface of the left forearm. The bent forearm was covered with black cardboard. Visual comparison stimuli and fixation crosses were projected on the cardboard. Subjects were asked to press a left (right) button when they felt the tactile standard left (right) of the visual comparison.

A schematic of the experimental setup is depicted in Figure 2. Subjects sat in front of the apparatus which was mounted on a table. The left forearm was placed inside the apparatus, parallel to the torso. Three solenoids on a height-adjustable board were arranged directly above the arm. When an electrical current was applied to a solenoid it drove out a small pin (length: 9 mm, diameter: 1 mm) which gently touched the dorsal surface of the arm. Touches (*tactile standard stimuli*) were located at 10° left, 10° right, and central (0°) to the right eye and with the midpoint of the arm (from elbow to wrist) roughly aligned with the central stimulus. The distance between the right eye and the central stimulus was approximately 25.5 cm.

To mask the sounds associated with touch presentation, subjects wore in-ear headphones (Philips SHE8500) presenting white noise.

The arm and the solenoids were covered with a horizontally mounted black cardboard on which the visual comparison and fixation stimuli were projected by an LCD projector. Before each session a calibration grid was projected directly on the tactile stimuli (cardboard was removed) that were fixed within the apparatus in order to ensure that tactile and visual presentations were aligned. *Visual comparison stimuli* were single white dots (diameter: 5 mm) varying in location between 25° left and 25° right of each standard location. For each trial, the location of the visual comparison stimulus was determined by an adaptive staircase procedure with variable step size (see section Adaptive Staircase Procedure).

Fixation stimuli consisted of a white cross (height/length: 10 mm) which was presented 20° left or right of the location of the tactile standard stimuli. To ensure compliance with instructions, we recorded movements of the right eye by a head mounted EyeLinkII eye tracker system (SR Research) at a sampling rate of 250 Hz. Before each experimental block the eye tracker was calibrated with a horizontal three point calibration at 10° left, 10° right and 0° (the tactile standard locations). Responses were given by left or right button presses. Participants performed the task in a dark room. To avoid dark adaptation a small halogen table lamp was switched on for 800 ms before every trial. The experiment was performed using Presentation® software (Version 15.0, www.neurobs.com).

### Procedure

Subjects completed a spatial localization task (yes/no paradigm) by indicating if they perceived a tactile standard stimulus left or right of a visual comparison stimulus. The task was performed under two gaze conditions (*fixed* vs. *shifted*) and two orders of stimulus presentation (*vis-tac* vs. *tac-vis*).

#### Adaptive staircase procedure

Each standard-fixation combination (−10°/0°/10° × −20°/20°) was performed with two opposing staircases which differed by the initial location of the visual comparison. While one staircase started with an initial position at 25° left, the other started at 25° right to the standard location. Within each staircase, an adaptive algorithm determined both the magnitude of the shift of the visual comparison for the next trial (the step size) and the direction in which the visual comparison was shifted depending on the subject's response in the previous trial. More specifically, the step size was gradually decreased while the visual comparison approached the perceived standard location and thus, placing more observations around the parameter of interest (PSE).

**Figure 3:**
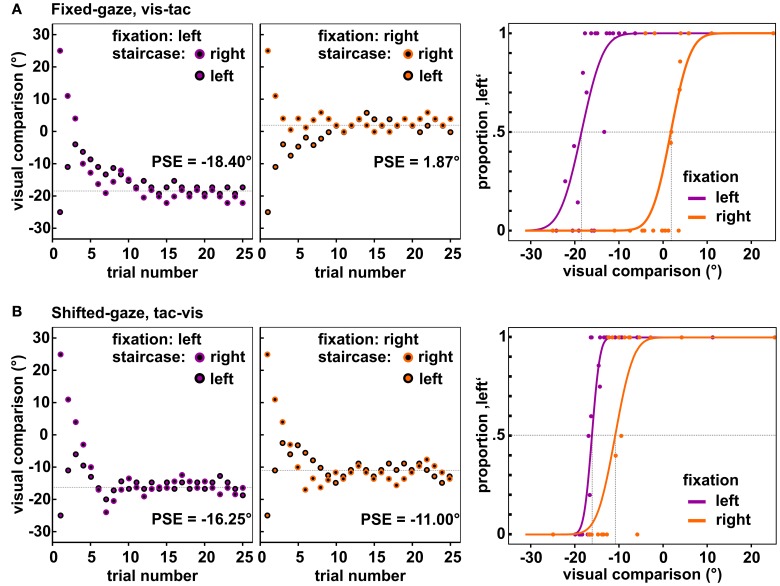
**Observed data of one subject for the central standard location**. Depicted are the locations of the visual comparison presented in each trial of the two staircases when fixating to the left (1st panels) and to the right (2nd panels). Each staircase comprised 25 trials, resulting in 50 trials per fixation-standard combination. Over the course of trials the two staircases approached the PSE (dashed line). The 3rd panel in each row shows the resulting psychometric functions fitted to the responses collapsed across the two opposing staircases. **(A)** Data of the condition fixed-gaze, *vis-tac* for which we expected a differential influence of fixation on the visual and tactile stimulus, i.e., a significant difference between the PSEs for the left and right fixation (vertical dashed lines). **(B)** Data of the condition shifted-gaze, *tac-vis* for which we expected the same influence of gaze on both stimulus modalities, i.e., no significant difference between the PSEs (vertical dashed lines).

The applied algorithm consisted in the accelerated stochastic approximation developed by Kesten (1958) and implemented a reduction of step size for each time the response (left or right) changed with respect to the preceding trial within one staircase. The initial step size was set to 28° which was reduced to half before the first step was carried out, i.e., the largest possible step size was 14° (for details, see Treutwein, 1995). The minimal step size was set to 2°. The direction of each step depended on the previous response within the respective staircase and placed the visual comparison closer to the perceived standard location; e.g., if the subject indicated that the tactile standard was left (right) to the visual comparison, in the next trial the visual comparison was shifted leftwards (rightwards). We further imposed the restriction on the first trial within each staircase (where the visual comparison was separated from the standard by 25°) that it had to be classified correctly; otherwise the first trial was repeated. Distances were set on the basis of previous findings that were obtained with a comparable setup (Mueller and Fiehler, 2013). Figure 3 displays exemplary data obtained from one subject for both fixations and the two gaze conditions [panel (A) and panel (B)].

#### Gaze conditions

**Figure 4:**
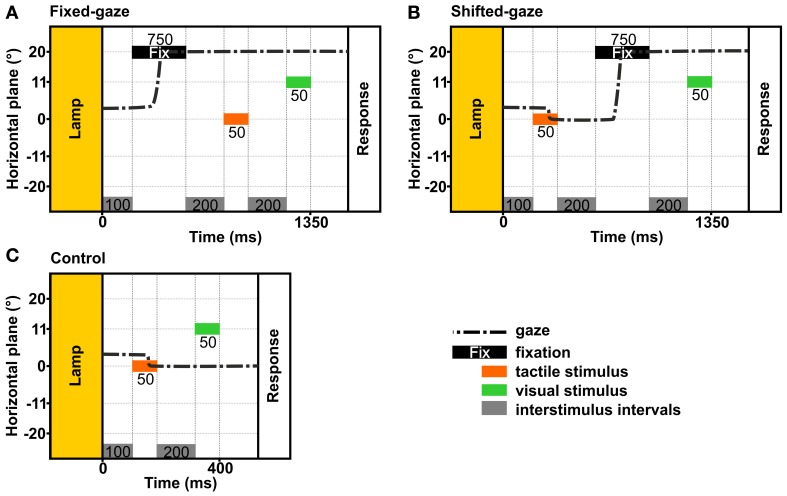
**Temporal schematics of the gaze conditions**. The examples show the trial timing in the stimulus order *tac-vis* for the central standard location and the right fixation. In each condition, gaze was held at a previously indicated location until the response was given. **(A)** Fixed-gaze: gaze was aligned with the fixation location before the tactile standard and the visual comparison were presented sequentially. **(B)** Shifted-gaze: subjects foveated the tactile standard, then shifted gaze toward the fixation location where it was held while the visual comparison was presented. **(C)** Control: subjects foveated the tactile standard and held gaze at this location while the visual comparison was presented.

In order to examine how an eye movement intervening stimulus presentation and response affects the reference frame of tactile and visual stimulus localizations, we applied two gaze conditions: (a) a fixed-gaze condition and (b) a shifted-gaze condition (see Figure 4). In both gaze conditions, gaze was directed at a fixation location during the response. In every trial, the tactile standard and the visual comparison were presented for 50 ms each.

The *fixed-gaze condition* (Figure 4A) started with the presentation of the fixation cross for 750 ms. Subjects were asked to fixate the indicated location and to maintain gaze at this location until they delivered the response. Thereby, gaze was fixed while the standard and the comparison stimulus were presented.

The *shifted-gaze condition* (Figure 4B) began with the presentation of the first stimulus; this could be either the standard or the comparison stimulus depending on stimulus order. Subjects were asked to fixate the felt or viewed location of the first stimulus before they shifted gaze toward the fixation location as soon as the fixation cross was presented. Gaze had to be held at the fixation location until the response was given. Thereby, an eye movement was introduced between the first and the second stimulus (i.e., between the presentation of the tactile standard and the visual comparison); thus the second stimulus was presented while gaze was directed at the fixation location.

#### Stimulus order

To examine the differential effects of a gaze shift on the two stimulus modalities we varied the order in which they were presented. In the *vis-tac condition*, the visual comparison was presented before the tactile standard. In the *tac-vis condition*, the tactile standard was presented before the visual comparison stimulus. Note that depending on the gaze-condition gaze was aligned with the fixation location *before* or *between* the presentation of the standard and the comparison.

#### Control condition

**Figure 5:**
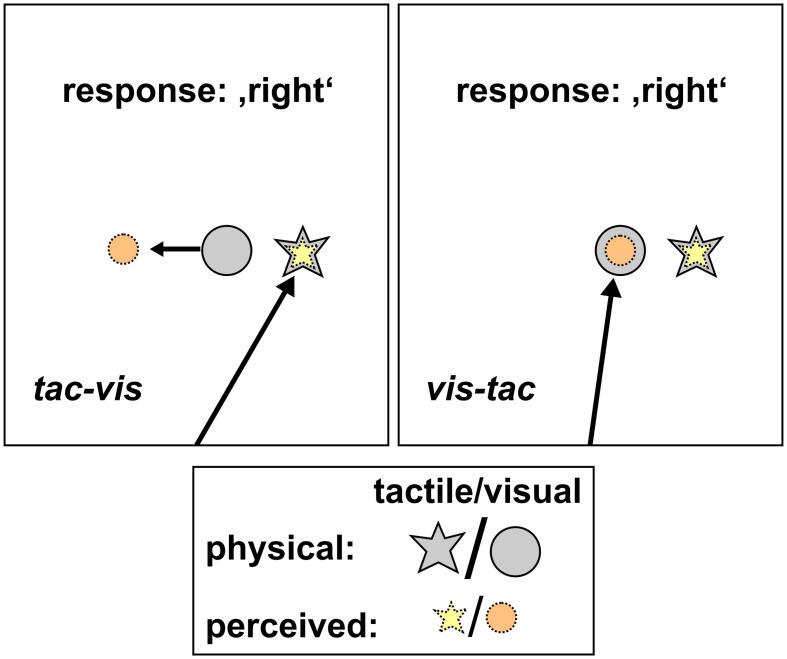
**Schematic hypotheses of the control condition for both stimulus orders**. *Tac-vis* **(left panel)**: subjects fixated the perceived location of the tactile standard (yellow star) and held gaze at this location while the visual comparison (gray circle) was presented subsequently. Viewing the visual comparison peripherally should lead to a misestimation of its position opposite to gaze direction (orange circle), however, leaving the left/right judgment unaffected. *Vis-tac* **(right panel)**: subjects fixated the visual comparison (gray circle) and held gaze at this location while the tactile standard (gray star) was presented subsequently. The foveated visual stimulus should be localized accurately (orange circle), thus providing a spatially correct reference when judging the location of the tactile standard (yellow star).

We further applied a control condition (Figures 4C, 5) where subjects were asked to fixate the first stimulus and keep gaze at this location until the response. This condition was introduced to assess the perceived location of the tactile standards while gaze was either maintained at the standard (*tac-vis*, Figure 5, left) or held eccentric to the standard (*vis-tac*, Figure 5, right); i.e., no gaze shift occurred between the presentation of the standard and the comparison. For neither stimulus order we expect an influence of gaze direction biasing the spatial judgment of the tactile standard stimulus.

### Data analysis

We assessed PSEs as a function of fixation (left or right) depending on gaze condition and stimulus order.

Two opposing staircases (starting 25° left/right of the standard, see Figure 3) were conducted for the three standard locations (10° left/right and central) combined with the two fixations (20° left/right of the standard), resulting in 12 staircases (2 × 3 × 2) for each gaze condition (fixed/shifted). The 24 staircases were performed in two stimulus orders (*vis-tac/tac-vis*), totaling in 48 staircases. The control condition did not involve different fixations reducing the number of staircases to 6 (2 × 3) which were carried out for the two stimulus orders, i.e., in total 12 staircases. Each staircase comprised 25 trials. Trials were randomized across the staircases within each gaze condition and within the control condition. Every 100 trials short breaks, with the light turned on, were included.

Stimulus order was varied in separate sessions and counterbalanced across participants. Within each stimulus order, gaze and control conditions were performed in randomized order. For data analyses, we collapsed the two opposing staircases that belonged together.

Eye tracking data were exported into a custom graphical user interface (GUI) written in MatLab R2007b (TheMathWorks Inc., Natrick, MA) to ensure subjects' compliance with instructions in every trial. Trials were classified as valid and included in data analyses if gaze stayed within ±2.5° degree of the fixation location until the response was recorded. The percentage of valid trials had to be higher than 60% in every condition, otherwise the subject was excluded, yielding 10 (out of 15) remaining subjects for further analyses.

For valid trials, psychometric functions were fitted using psignifit version 2.5.6 (see http://bootstrap-software.org/psignifit/), a software package which implements the maximum-likelihood method described by Wichmann and Hill (2001). In order to account for our sampling scheme where high intensity values were underrepresented we fixed gamma at 0. The fitting procedure was conducted separately for each participant and standard-fixation combination (−10°/0°/10° × *left/right*) in each condition (shifted/fixed gaze x *vis-tac/tac-vis* and control × *vis-tac/tac-vis*); totaling in 30 psychometric functions per subject. Supplementary Figure 1 depicts all psychometric functions of one subject in the two gaze conditions. The fitted parameter estimations for the PSE and the 84% difference threshold were exported to SPSS (SPSS Inc., Chicago, IL) wherewith all further computations were performed.

### Statistical analyses

We conducted a cross-modal spatial localization task in which the location of a remembered tactile stimulus had to be judged as left or right to a remembered visual comparison stimulus.

In order to check whether participants were able to discriminate the three standard locations, we first analyzed the PSEs of the control condition with a Two-Way RM ANOVA [standard location (3) × stimulus order (2)]. Analogously, we analyzed the slopes of the control condition indicating the precision of the spatial judgments.

To test our hypothesis (see Figure 1) that PSEs vary as a function of fixation depending on both stimulus order and gaze condition (Three-Way interaction), we conducted a 2 × 2 × 2 repeated measures analysis of variance (RM ANOVA) on PSEs with the factors gaze condition (*fixed/shifted*), stimulus order (*vis-tac*/*tac-vis*) and fixation (*left/right*).

Second, we analyzed PSE shifts as a function of fixation depending on stimulus order within each gaze condition by conducting a 2 × 2 RM ANOVA with the factors gaze condition and fixation. According to our hypothesis (see Figure 1), we expected a main effect of fixation in the fixed-gaze condition and an interaction between stimulus order and fixation in the shifted-gaze condition. To further examine a putative interaction in the shifted-gaze condition, one-tailed paired *t*-tests are performed to test for significant differences between the left and right fixation (PSE_left_ < PSE_right_) within each stimulus order. We expect PSEs to significantly differ across fixations for the *vis-tac* but not for the *tac-vis* condition.

Finally, we analyzed the precision of the spatial judgments as a function of stimulus order, gaze condition, and fixation [RM ANOVA: stimulus order (2) × gaze condition (2) × fixation (2)]. However, the conclusive value of this analysis is restricted by the fact that the applied adaptive algorithm aimed to estimate the PSE and not the slope (see Levitt, 1971, for details on the features of psychophysical procedures).

Each time sphericity was violated as determined with Mauchly's test, Greenhouse-Geisser corrected *p*-values are reported.

## Results

The present study aimed to examine how a gaze shift after the presentation of a tactile target changes its reference frame. Based on the assumption that spatial localization and goal-directed movements to targets share similar spatial coding mechanisms, we expect a switch from a gaze-independent to a gaze-dependent reference frame for tactile targets in a visuotactile spatial localization task, consistent with our previous findings on goal-directed reaching to tactile targets (Mueller and Fiehler, 2013).

### Control condition

In order to assess the perceived location of the tactile standard in the absence of a bias with respect to gaze direction (see Figure 5), we conducted a control condition where participants were asked to fixate the first stimulus which could either be the tactile standard or the visual comparison depending on stimulus order (*tac-vis/vis-tac*). For the stimulus order *tac-vis*, subjects fixated the perceived location of the tactile standard and judged its relative location by simply indicating if it was left or right of the visual comparison that was subsequently presented into the visual periphery. Even if the visual stimulus was shifted with respect to gaze, it should not change the subject's response and thus, the PSEs (see Figure 5, left). For the stimulus order *vis-tac*, we assume that the fixated location of a visual stimulus can be judged quite accurately (Bock, 1986; Henriques et al., 1998) and therefore should provide a veridical reference when judging the location of the tactile stimulus which was subsequently presented (see Figure 5, right).

**Figure 6:**
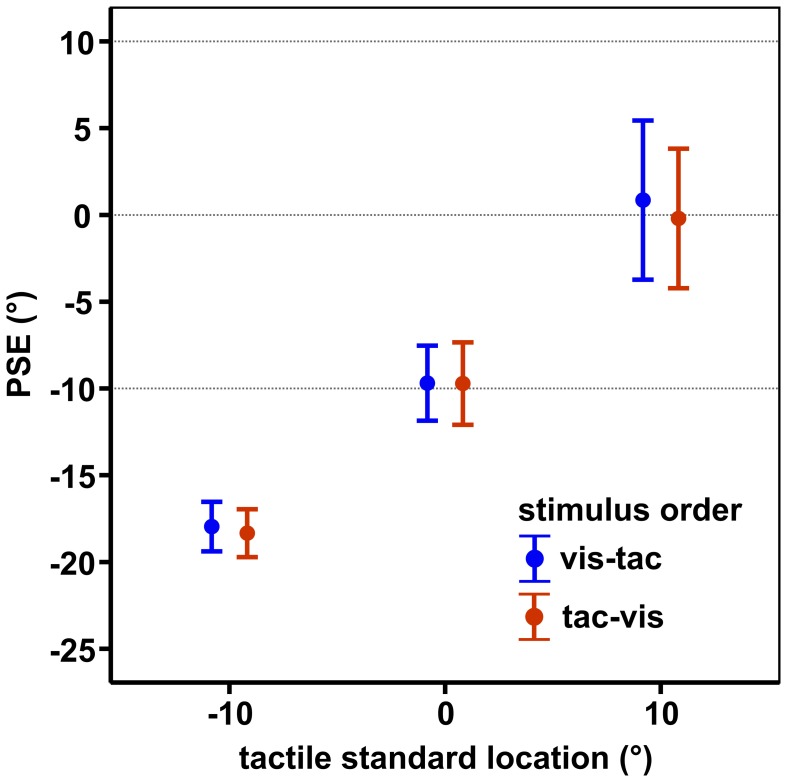
**Mean PSEs of the control condition, averaged across subjects for each standard location and stimulus order**. Error bars display the standard errors of the mean. Horizontal dashed lines indicate the physical standard locations.

Results are shown in Figure 6. Mean PSEs (see Table 1) significantly varied with standard location irrespective of stimulus order [main effect standard location: *F*_(2, 18)_ = 23.7, *p* = 0.001], indicating that subjects were able to discriminate the three touch locations. As reported in previous studies (Harrar and Harris, 2009; Jones et al., 2012; Mueller and Fiehler, 2013), we observed a constant bias toward the side of the body where the limb was stimulated, i.e., when a somatosensory target is on the left hand or arm it is felt more leftward than it actually is. However, the magnitude of mislocalization reported in the literature (Harrar and Harris, 2009; Jones et al., 2012) is on average smaller (about 2 cm) compared to our results (about 4 cm). Since we observed similar (gaze-independent) biases in another experiment conducted with this setup (Mueller and Fiehler, 2013) we consider the increased magnitude of biases as reflecting a peculiarity of the setup which does not vary across conditions.

**Table 1:** **Mean PSEs and slopes with standard errors of the means of the control condition**.

	**Stimulus order**	**Tactile standard location**		
		−**10°**	**0°**	**10°**
PSEs	***vis-tac***	−18.02 ± 1.45	−9.72 ± 2.16	0.74 ± 4.60
	***tac-vis***	−18.34 ± 1.38	−9.86 ± 2.39	−0.38 ± 3.95
Slopes	***vis-tac***	5.16 ± 1.31	5.15 ± 1.32	4.79 ± 1.01
	***tac-vis***	4.73 ± 0.96	3.74 ± 0.69	5.60 ± 1.20

*Data were averaged across subjects for each tactile standard location and stimulus order*.

Slopes did not vary with standard location (−10°/0°/10°) or stimulus order (*vis-tac*/*tac-vis*) in the control condition (*p*'s > 0.05; Table 1). Therefore, for further analyses we collapsed the data across the three standard locations.

### Influence of gaze on tactile localization

**Figure 7:**
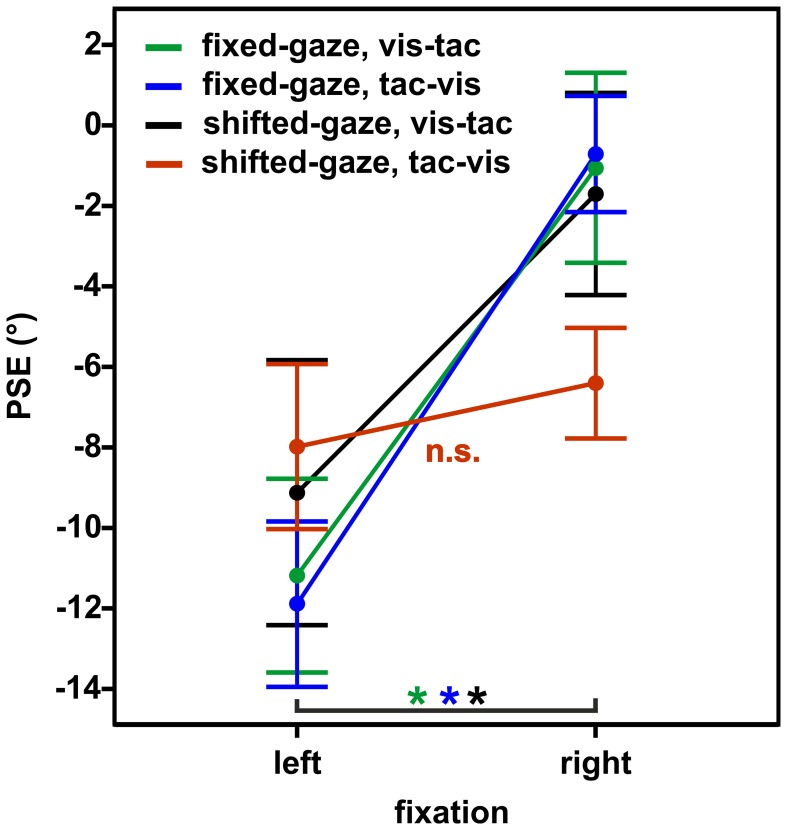
**Mean PSEs as a function of fixation, averaged across subjects for the experimental conditions (gaze condition × stimulus order)**. Error bars display the standard errors of the mean.

We conducted a Three-Way RM ANOVA with the factors stimulus order (2) × gaze condition (2) × fixation (2) on PSEs. We expected a different effect of *fixation* on PSEs in the condition where gaze was shifted *after* the encoding of the tactile standard (shifted-gaze, *tac-vis*) compared to the conditions where gaze was *fixed* (fixed-gaze, *tac-vis* and fixed-gaze, *vis-tac*) or gaze was shifted *before* the tactile standard was presented (shifted-gaze, *vis-tac*), resulting in a Three-Way interaction of stimulus order, gaze condition and fixation. Indeed, gaze condition interacted with fixation depending on the level of stimulus order [Three-Way interaction: *F*_(1, 9)_ = 21.6, *p* = 0.001]. Figure 7 displays the mean PSEs as a function of fixation (x-axis) for each stimulus order combined with each gaze condition. To further explore this effect of gaze, we examined the interaction for fixed- and shifted-gaze, separately.

In the fixed-gaze condition, we expected the PSEs to vary as a function of fixation due to a gaze-dependent mislocalization of the visual comparison but not of the tactile standard (represented in a gaze-independent frame) as illustrated in Figure 1A. This effect should occur irrespective of the order in which the standard and the comparison stimuli were presented. Consistent with our hypothesis, we found a main effect of fixation [*F*_(1, 9)_ = 20.3, *p* = 0.001; Figure 7, green and blue line) that did not vary with stimulus order [interaction: *F*_(1, 9)_ = 0.2, *p* = 0.650].

In the shifted-gaze condition, we hypothesized that the effect of fixation would critically depend on the order in which the tactile standard and the visual comparison were presented. Specifically, we expected that a gaze shift *after* the presentation of the tactile standard would trigger a shift from a gaze-independent to a gaze-dependent representation of the tactile standard. This, in turn, should result in a predominantly gaze-dependent representation of both the tactile standard and the visual comparison reflected by similar PSEs. That means, the effect of fixation should be comparable for the tactile standard and the visual comparison, thereby keeping their spatial relation constant (see Figure 1B). In accordance with our hypothesis, PSEs varied as a function of fixation depending on stimulus order [interaction: *F*_(1, 9)_ = 15.4, *p* = 0.003]. We further explored this effect by calculating *post-hoc* paired *t*-tests. The results demonstrated that PSEs significantly differed as a function of fixation [*t*_(9)_ = −6.0, *p* < 0.001] if gaze was shifted from the visual comparison to the fixation location *before* the tactile standard was presented (stimulus order *vis-tac*; Figure 7, black line). However, if gaze was shifted *after* the encoding of the tactile standard, this effect vanished [*t*_(9)_ = −1.2, *p* = 0.270; Figure 7, red line].

To check for putative effects caused by the three different standard locations (−10°/0°/10°) we further performed the respective paired *t*-tests separately for the three individual touch locations using Bonferroni-adjusted alpha levels of *p* < 0.008 (0.05/6). Results were confirmed for each of the standard locations with significantly smaller PSEs for fixations to the left than to the right in the *vis-tac* condition (*p*'s < 0.005) but not in the *tac-vis* condition (*p*'s > 0.063).

### Slopes

To test for differences in precision, the slopes of the psychometric functions were analyzed. We conducted a Three-Way RM ANOVA for stimulus order (2) × gaze condition (2) × fixation (2) analog to the analysis performed on the PSEs, and obtained an interaction between gaze condition and fixation [*F*_(1, 9)_ = 8.8, *p* = 0.016]. We further explored this effect by testing the difference within each gaze condition between the left and right fixation as well as the difference across gaze conditions within each fixation (averaged across stimulus orders). *Post-hoc* paired *t*-tests yielded no significant differences (*p*'s > 0.076).

## Discussion

The present study investigated the role of an effector movement (gaze shift) on spatial coding and updating of tactile stimuli in a gaze-dependent reference frame. To this end, we examined how the spatial relation of a tactile and a visual stimulus varied with gaze direction (fixation *left/right*) depending on stimulus order (*vis-tac/tac-vis*) and gaze condition (fixed/shifted) in a visuotactile spatial localization task (yes/no paradigm). We found that gaze direction similarly influenced the localization of both the tactile and the visual stimulus when a gaze shift occurred after the presentation of the tactile stimulus (shifted-gaze, *tac-vis*). In contrast, when gaze was fixed at an eccentric location before the tactile stimulus was presented (shifted-gaze, *vis-tac* and fixed-gaze, *tac-vis/vis-tac*) gaze direction differentially affected the spatial localization of the tactile and the visual stimulus.

The present results support our previous findings obtained in a goal-directed reaching task where we observed gaze-dependent reach errors when subjects either moved their eyes or arm/hand (effector movement) before they reached to a somatosensory (tactile or proprioceptive-tactile) target in comparison to conditions where no effector movement occurred (Mueller and Fiehler, 2013). This finding suggests a switch from a gaze-independent to a gaze-dependent spatial representation of remembered tactile stimuli triggered by an effector movement. Because the positional judgment task, used here, required no reaching movement we can rule out that the observed bias in spatial localization is due to proprioceptive mislocalization of the reaching hand (cf., Dessing et al., 2012), strengthening our previous findings (Mueller and Fiehler, 2013). Instead, the obtained biases rather reflect a mislocalization of the remembered target opposite to gaze direction (cf., Bock, 1986; Henriques et al., 1998).

Since we assessed the *relative* location of two subsequently presented stimuli in a cross-modal task, different hypotheses about the relative mislocalization of the tactile and the visual stimulus can be generated. Based on a considerable amount of research on spatial coding and updating of visual targets [localization tasks: (Lewald and Ehrenstein, 1996, 1998; Eggert et al., 2001; Fiehler et al., 2010); goal-directed reaching tasks: (Henriques et al., 1998; Lewald, 1998; Jones and Henriques, 2010)], we assume that the location of the visual comparison was always overestimated in the opposite direction of gaze. Following this assumption, we are able to infer the perceived location of the tactile stimulus by interpreting the positional judgments of the tactile standard relative to the visual comparison, expressed by the PSE. We interpret similar PSEs for both fixations as evidence for a gaze-dependent spatial representation of both visual and tactile stimuli while differences in PSEs are taken as evidence for a gaze-dependent representation of the visual but not of the tactile stimulus. We are aware that the PSEs differing between fixations could also be explained by opposing localization errors of visual (opposite to gaze) and tactile (in the direction of gaze) stimuli (cf., Harrar and Harris, 2009, 2010). The direction of gaze-dependent localization errors of tactile stimuli (in the direction or opposite to gaze) seems to depend on the task, in particular on head eccentricity during the time of response (Pritchett et al., 2012). However, opposing error patterns are unable to explain similar PSEs for both fixation sides, as we found for the condition where an effector movement (gaze shift) occurred after tactile stimulus encoding.

While tactile spatial information enters the nervous system in somatotopic coordinates unaffected by gaze direction, a gaze shift after the encoding of the tactile stimulus seems to trigger an update of its remembered location in gaze coordinates. Pritchett et al. (2012) also observed a switch from a body-centered to a gaze-centered reference frame when participants turned their head with the eyes (gaze = head angle + eye angle) after target presentation and before reporting the touch location on a visual scale. In line with the present findings, they concluded that tactile targets are coded in a gaze-centered reference frame “when the locations of the touches need to be remembered and reconstructed after a move.” These findings together with previous studies on gaze-dependent spatial updating of visual and proprioceptive targets (Henriques et al., 1998; Pouget et al., 2002b; Beurze et al., 2006; Fiehler et al., 2010; Jones and Henriques, 2010; Reuschel et al., 2012; Schütz et al., 2013) indicate that spatial updating seems to be a mechanism which operates in gaze-centered coordinates irrespective of the modality by which the location was originally perceived. However, it does not exclude a contribution of additional non-retinotopic reference frames, not tested here (cf., Pouget et al., 2002a). The use of a shared gaze-centered representation might facilitate the integration of spatial information from different sensory modalities, especially in situations where an effector movement requires a fast and continuous update of information in space. Electrophysiological studies in monkeys have demonstrated that gaze-centered spatial updating is based on predictive signals of neurons in the posterior parietal cortex which provoke a shift of visual receptive fields to the new updated location even 80 ms before the beginning of the eye movement (Duhamel et al., 1992). Little is known about predictive spatial updating of tactile receptive fields. Avillac et al. (2005) determined the reference frame of tactile targets (air puffs) in area VIP of the posterior parietal cortex while the monkey fixated one of three visual targets. They found that eye position did not affect tactile receptive fields suggesting spatial coding in head/body-centered coordinates, consistent with our results in the conditions where gaze was held at an eccentric location before the tactile stimulus was encoded (fixed-gaze, vis-tac/tac-vis and shifted-gaze, vis-tac). So far (at least to our knowledge), studies investigating spatial updating of tactile receptive fields triggered by a gaze shift are lacking.

Further evidence for gaze-centered spatial updating comes from research on goal-directed reaching where an influence of gaze shifts on reach endpoints has been reported for visual (for reviews see, Medendorp et al., 2008; Medendorp, 2011), auditory (Pouget et al., 2002b), proprioceptive (Pouget et al., 2002b; Jones and Henriques, 2010; Reuschel et al., 2012) and tactile (Buchholz et al., 2013) targets. Together with the present findings on tactile spatial localization, these findings suggest a similar underlying reference frame for spatial perception and goal-directed movements. The use of a common frame of reference may facilitate the interaction of space perception and action; two functions that are tightly coupled at the behavioral and neuronal level.

In sum, our results suggest that an intervening effector movement (gaze shift) changes the reference frame of tactile targets in a spatial localization task. While spatial information about visual and tactile stimuli enters the nervous system through different sensory channels associated with different reference frames, it is updated in gaze-centered coordinates triggered by an intervening gaze shift. This mechanism seems to apply for goal-directed reaching (Mueller and Fiehler, 2013) as well as for spatial localization.

## Author contributions

Stefanie Mueller and Katja Fiehler designed the experiment, Stefanie Mueller collected and analyzed the data, Stefanie Mueller and Katja Fiehler wrote the paper.

### Conflict of interest statement

The authors declare that the research was conducted in the absence of any commercial or financial relationships that could be construed as a potential conflict of interest. The Associate Editor, Knut Drewing, declares that, despite being affiliated with the same institution as the authors, Stefanie Mueller and Katja Fiehler, the review process was handled objectively and no conflict of interest exists.

## Supplementary material

Supplementary Figure 1 **Psychometric functions of one exemplary subject for the fixedand shifted-gaze conditions**.
