# Supplementary material for: Gaze-dependent spatial updating of tactile targets in a localization task
Source: Front Psychol. 2014 Feb 10;5:66. doi: 10.3389/fpsyg.2014.00066 (PMC3918658; doi:10.3389/fpsyg.2014.00066)
Supplement: Supplementary Figure 1 — Psychometric functions of one exemplary subject for the fixedand shifted-gaze conditions. [file DataSheet1.PDF]

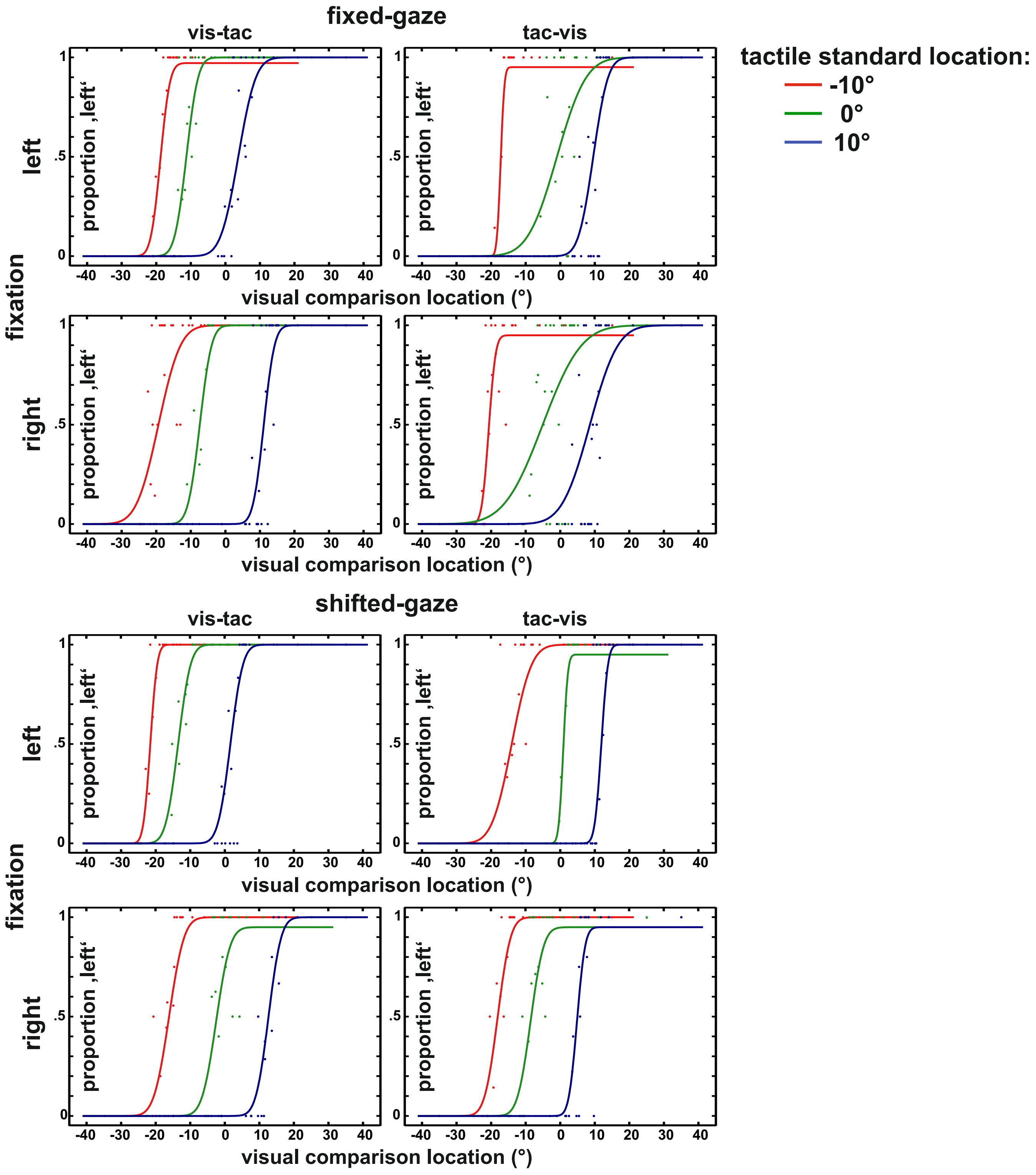

Supplementary Figure 1. Psychometric functions of one exemplary subject for the fixed- and shifted-gaze conditions.
